# From deficiency to excess: the impact of iodine excess on reproductive health

**DOI:** 10.3389/fendo.2025.1568059

**Published:** 2025-04-30

**Authors:** Aiman Khudair, Ahmed Khudair, Sara Anjum Niinuma, Haniya Habib, Alexandra E. Butler

**Affiliations:** ^1^ School of Medicine, Royal College of Surgeons in Ireland - Medical University of Bahrain, Busaiteen, Bahrain; ^2^ Research Department, Royal College of Surgeons in Ireland - Medical University of Bahrain, Busaiteen, Bahrain

**Keywords:** iodine, iodine excess, thyroid, thyroid hormones, reproductive health, infertility

## Abstract

Iodine is a critical trace element in the human body. It is primarily obtained through dietary sources such as dairy products, seafood, fish, eggs and certain vegetables. Iodine plays an essential role in various bodily functions, most notably in producing the thyroid hormones, triiodothyronine and thyroxine. Additionally, it influences the immune, cardiovascular, reproductive and gastrointestinal systems. Historically, iodine deficiency has been a significant global health issue; however, over the past decade, there has been a rise in iodine excess. This surge has been primarily attributed to inadequate monitoring and over-iodization of salt. Despite the well-documented consequences of iodine deficiency, the ramifications of excessive iodine intake remain underexplored. In view of rising global infertility rates, excess iodine has been linked to significant reproductive health effects. These include decreased sperm count, motility and morphology in males, as well as adverse pregnancy outcomes in females, such as maternal thyroid dysfunction and congenital hypothyroidism. This mini-review aims to collate and analyze current literature pertaining to the effects of iodine excess on reproductive health and shed light on its increasing incidence worldwide. Further research on the biological and clinical effects of iodine excess is required to derive a better understanding of this issue. Given the rising prevalence of iodine excess, it is crucial to raise awareness and implement proactive measures to prevent it from escalating into a major public health crisis in the future.

## Introduction

### What is iodine

Iodine is a trace element in the human body, found at levels of 15-20 mg in a healthy adult, with the majority (70-80%) contained within the thyroid gland; it is acquired solely through dietary sources (1–7). Iodine is naturally present in foods such as dairy, seaweed, fish, eggs, broccoli, spinach and peas. Iodine plays several roles in the human body, primarily in the synthesis of thyroid hormones such as triiodothyronine (T_3_) and thyroxine (T_4_) (1). This element is implicated in thyroid function and exerts profound effects on immune system modulation, the cardiovascular and reproductive systems, and gastrointestinal disorders, among others (1, 8, 9).

## Daily recommended intake

The daily recommended intake for iodine according to the World Health Organization (WHO) is different for schoolchildren, at 120 µg, versus adults at 150 µg, with increased levels recommended for pregnant/lactating women at 250 µg (10–12). Historically, iodine deficiency has been a major concern, prompting countries such as the United States and Switzerland to introduce iodized table salt in the 1920s, which helped improve iodine levels (1, 13, 14). By the 1990s, these salt iodization programs were adopted globally, leading to a decrease in the prevalence of iodine deficiency worldwide (1, 13, 14). As of 2019, global iodine deficiency rates were at 2.4% (14).

### Search strategy

A literature search was conducted between October 2024 to January 2025 utilizing Google Scholar and PubMed databases. The search employed keywords, including “iodine excess”, “iodine toxicity”, and “iodine overload”, among others. Only articles written in the English language were considered. Articles were included based on relevancy.

#### From deficiency to excess

Within the past decade, the number of countries with iodine deficiency has reduced from 54 to 30, while those with adequate levels have increased from 67 to 112 (15). Meanwhile, the number of countries with excessive iodine intake (defined as a median urinary iodine level over 300 µg/L) has doubled from 5 to 10: Brazil, Colombia, Somalia, Uganda, Armenia, Benin, Georgia, Honduras, Paraguay, Uruguay (15). Poor monitoring and over-iodization of salt have been the primary factors contributing to excessive iodine levels in these countries (15). The American Thyroid Association (ATA) sets the upper tolerable limit of iodine at 1100 µg and advises against daily intake exceeding 500 µg for children, adults and pregnant or lactating individuals (16). Meanwhile, the Joint Food and Agriculture Organization (FAO)/World Health Organization (WHO) Expert Committee on Food Additives (JECFA) has proposed a maximum upper limit of 1 mg/day of iodine for the general population (10–12). Other organizations such as the former Scientific Committee on Food (SCF) of the European Commission and the UK Expert Group on Vitamins & Minerals (EVM) propose upper tolerable limit values for adults at 600 µg/day and 500 µg/day respectively (17, 18).

It is important to recognize that individuals can differ significantly in terms of their tolerance to iodine intake due to a multitude of factors. Diet plays an important role, as varying amounts and sources of iodine with differing eating patterns contribute to inter-individual variability (19). Furthermore, those with pre-existing thyroid disease may be more susceptible to excessive iodine intake (10). Genetic mutation can additionally impact the normal physiological processes of the thyroid to iodine (20). Consequently, these factors should be considered when interpreting studies.

Although the impact of iodine deficiency is well-studied, iodine excess has received much less attention regarding its effects on human health. The literature contains a paucity of articles assessing the impacts of excess iodine especially in terms of reproductive health; however, some studies have shown excess iodine affects reproductive health and pregnancy by impairing spermatogenesis, hindering infant neurodevelopment, increasing the risk of infertility and contributing to gestational diabetes mellitus (GDM) and hypertensive disorders during pregnancy (HDP) (21–24). In this mini-review, we aim to highlight the impact excess iodine has on the reproductive health of males and females.

#### The body’s reaction to excessive iodine exposure

Historically, one of the earliest mentions of the reaction of the thyroid gland to acute excessive iodine concentrations was described by Dr. Jan Wolff and Dr. Israel Chaikoff, an observation now known as the Wolff-Chaikoff effect (25, 26). By reducing the synthesis of T_3_ and T_4_, this effect acts to block the excessive amounts of iodide in the body from generating large amounts of thyroid hormone (27–29). One proposed mechanism of the Wolff-Chaikoff effect is the production of inhibitory substances that impact thyroid peroxidase, namely intrathyroidal iodoaldehydes, iodolactones or iodolipids (25, 30).

However, the Wolff-Chaikoff effect is transient, usually lasting only one to two days, after which an escape occurs where regular synthesis of thyroid hormones resumes (25, 27, 31). Reduced expression of the sodium-iodide symporter has been theorized to diminish iodide uptake and the subsequent generation of thyroid hormones, thereby maintaining a euthyroid state (25, 27, 31).

Notably, excess amounts of daily iodide ranging from 30 mg to 2 g are typically well tolerated in individuals with normal thyroid glands. Although laboratory alterations are seen, such as a reduction in serum T_4_ by 25%, T_3_ by 15% and a 2 mU/L rise in TSH, these values remain within the normal ranges without clinical signs of thyroid dysfunction (32).

A failure of the Wolff-Chaikoff effect can give rise to the Jod-Basedow phenomenon, typically seen in patients with impaired thyroid regulation, including those with thyroid nodules. Rather than becoming hypothyroid, these patients may generate an excessive amount of thyroid hormone (25, 28, 31, 33, 34).

Although the effect of iodine on thyroid pathophysiology is well described, the role that excess iodine has independent of thyroid hormone in regard to infertility has not been well documented (35). Infertility, defined as a couple’s inability to conceive after one year of regular unprotected intercourse, is a significant health issue impacting 50-80 million individuals worldwide (36–38). Infertility can be subcategorized into primary and secondary infertility, the former being when a woman has never conceived, and the latter being when a woman has had at least one successful previous conception but now has inability to conceive according to the WHO (39–41). Categories of infertility include ovulatory dysfunction, tubal occlusion, diminished ovarian reserve, endometriosis, uterine and male factors (42). Furthermore, lifestyle and environmental factors such as sexual violence, sexually transmitted diseases, stress and nutritional insufficiency among many others have also been associated with reduced fertility (43). An identifiable cause can be found in 85% of infertile couples, whereas the remaining 15% are classified as unexplained infertility (42, 44). Furthermore, infertility rates in men have been rising over the past decades, with a notable 76.9% increase since 1990 (45). Additionally, 20-30% of infertility cases are due to males, while male factors contribute to 50-70% of infertile couples (46). Therefore, it is imperative to highlight the possible role that excess iodine may have on exacerbating this global health crisis (47).

#### Iodine excess and its impact on male reproductive health

Recent studies have shown that excessive iodine levels correlate with several adverse effects on male reproductive health, particularly with regard to semen quality. A study investigating the association between semen quality and iodine intake in 1,098 fertile Chinese men found that participants with iodine excess, defined as a urinary iodine concentration (UIC) of ≥ 200 µg/L, exhibited a 5% higher semen volume, 26% lower semen concentration, 20% fewer semen counts, and a 1.48 month longer time to pregnancy in comparison to those with an optimal iodine intake (100 ≤ UIC < 200µg/L) (48). Although these findings were statistically significant, there was no significant effect on sperm motility (48). A strength of this paper is its relatively large multi-province sample of 1,098 couples, enhancing external validity; however, a limitation is its cross-sectional design which does not allow definitive establishment of causality. Furthermore, its sample inclusion of only fertile men, excluding infertile men, may underestimate the true effect of iodine on semen quality.

Another study conducted in Spain analyzing 96 couples with a median UIC of 97.6 µg/L undergoing infertility evaluation found a higher rate of altered spermatozoa morphology and a lower motile sperm count in those with higher semen iodine levels and urinary iodine levels respectively (35). Moreover, males who had not been able to achieve pregnancy with their partner for more than three years had a higher urinary iodine level than those trying for fewer than three years (35). In contrast to the previously mentioned study, this paper observed no association in certain semen characteristics such as volume, total sperm count and percentage of spermatozoa with progressive motility, after adjusting for age, smoking and BMI (35). Interestingly, the urinary iodine level was negatively correlated with the total motile sperm count (35). Moreover, males with abnormal sperm morphology possessed a higher median semen iodine level (35). Additionally, those who reported consuming iodized salt had significantly higher median semen iodine levels (16.5 µg/L) compared to those who consumed non-iodized salt (11.7 µg/L). A strength of this study is that it was the first study to measure semen iodine concentration in association with semen quality; a limitation is the small sample size. Although a causal relationship could not be established within the study due to cross-sectional design, the findings highlight the need for additional clinical research to investigate whether excess dietary iodine impacts spermatozoal morphology and other parameters, which could affect fertility.

The hypothalamic-pituitary-thyroid axis is key in reproductive health, especially in the context of iodine excess. Since iodine excess in certain susceptible individuals can lead to thyroid dysfunction (hypothyroidism or hyperthyroidism) (10). Hypothyroidism in humans can lead to reductions in total serum testosterone, luteinizing hormone (LH), follicle stimulating hormone, sex-hormone binding globulin and alterations in sperm quality (49–52). Similarly, adverse effects on fertility are seen in hyperthyroidism, such as alterations in sex steroid levels and spermatozoal DNA damage and motility abnormalities (53–55). Iodine excess’ effect on fertility is theorized to be a consequence of oxidative stress on the testis (53).

A randomized control trial studying the effects of iodine excess in adult male rats evaluated parameters including testicular morphology, steroidogenic enzyme activity, and sperm count, viability and morphology (56). They found that male rats administered 100 times and 500 times the recommended iodine level saw dose- and duration-dependent impairment of reproductive function compared with controls (56).

Interestingly, the role of iodine in male reproductive health is substantiated by the immunohistochemical confirmation of the sodium-iodide symporter and pendrin (57, 58). One proposed mechanism is reactive oxygen species (ROS) generation following excessive iodine accumulation within the testes, adjudged by an alteration in levels of pro-oxidant and antioxidants (catalase, superoxide dismutase, glutathione peroxidase) as well as increased levels of lipid peroxidation (56). Under normal physiological states, ROS is generated as part of the steriodogenesis process, however, as a result of iodine-induced alteration in antioxidant levels, this leads to the inability of the testes to counteract the harmful effects caused by ROS (56, 59) causing cellular damage to testicular germ cells (56, 60). Furthermore, this leads to a decrease in testosterone synthesis via inhibition of the enzymes Δ^5^ 3β-hydroxysteroid dehydrogenase (HSD) and 17 β-HSD (56). Since testosterone is an important regulator of spermatogenesis, these processes eventually result in functional and structural alterations in the testes (56, 61). Furthermore, prolonged ROS generation disrupts the hypothalamic-pituitary-adrenal axis by upregulating the adrenocortical stress signaling pathway thereby increasing corticosterone. This effect leads to a downstream inhibition of LH release further decreasing testosterone synthesis (56). Moreover, a protracted state of iodine excess can directly precipitate a hypothyroid state, eventually decreasing testosterone levels (56). While this study provides mechanistic insights, it was performed in rats, limiting translational relevance to humans.

Another study by Chakraborty et al. used rat models to evaluate the role of excess iodine in spermatogenesis. Similar to the previous findings, these authors observed a significant reduction in both sperm motility and sperm count (21). Furthermore, molecular investigations revealed that oxidative stress, increased apoptosis, reduced expression of blood-testis barrier (BTB) proteins, decreased regulators of spermatogenesis, and reduced expression of markers related to germ cell proliferation and differentiation all played a role in causing impaired spermatogenesis in the study (21). Ultimately, the mechanisms the authors propose that lead to spermatogenesis impairment is an interplay between cystoskeleton and BTB disruption as well as oxidative stress (21). Another paper confirmed that excess iodine, by inducing ROS generation, led to spermatozoal cell apoptosis thereby affecting male fertility (62).

**Figure 1:**
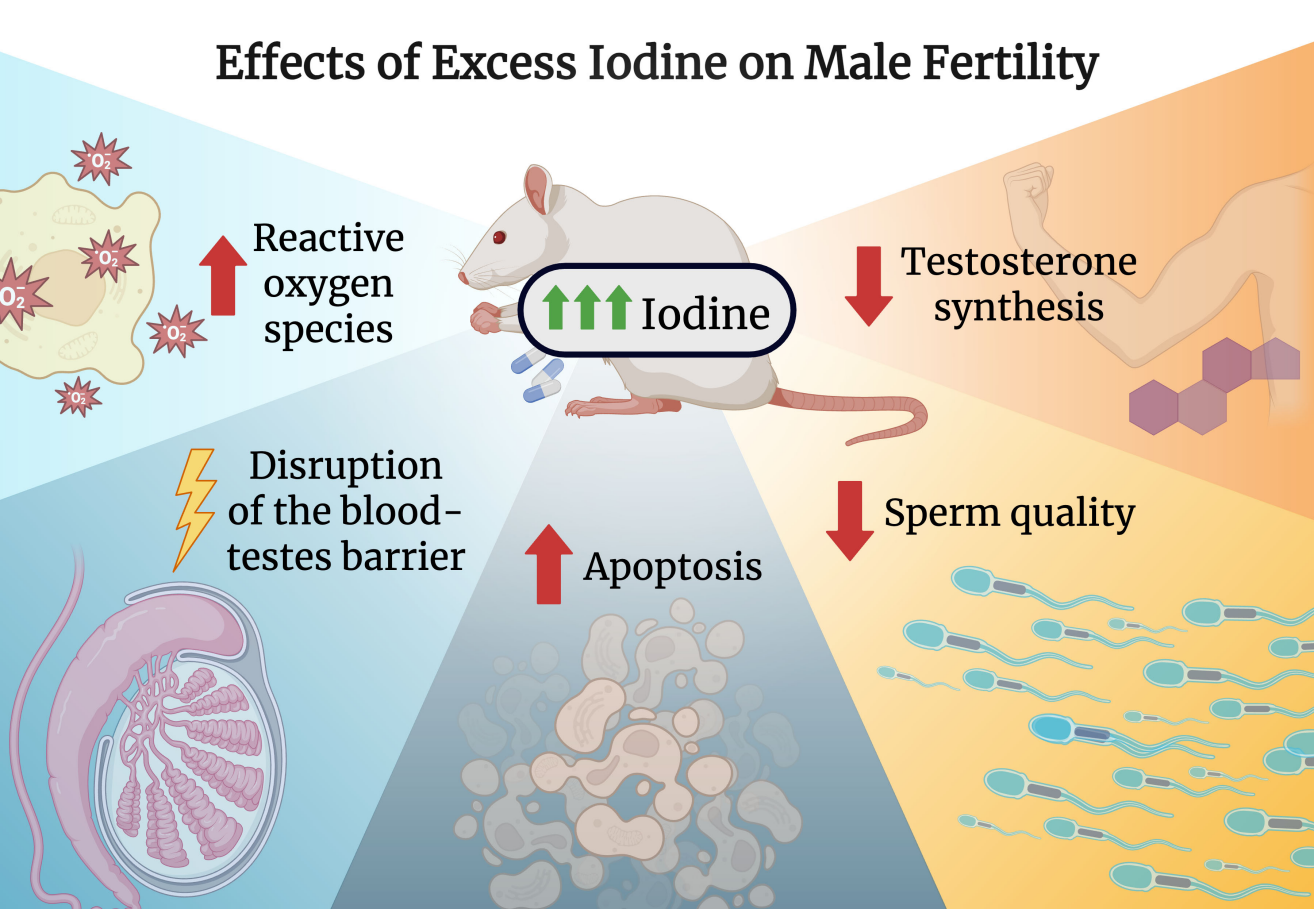
Effects of excess iodine on male fertility. Created with biorender.com.

Although these findings are based on rat models, they provide valuable insight into how iodine may impact human reproductive health (**Figure 1**). Hence, it is crucial to develop effective strategies to mitigate iodine-induced male infertility to combat the rise in infertility rates over the past decades.

## Iodine excess and its impact on female reproductive health

### Iodine excess and female fertility

Unexplained infertility (UI) is the diagnosis given when a couple has been actively trying to conceive for more than a year unsuccessfully, and medical testing cannot identify a cause for infertility (44). For women who have low fertility, hysterosalpingography (HSG) is historically the most common first-line diagnostic modality to assess the uterine cavity and tubal patency (63). However, a study using an oil-soluble contrast medium (OSCM) in conjunction with HSG caused iodine excess, leading to the development of subclinical hypothyroidism in approximately 40% of women, and later-onset hyperthyroidism in 5% of women (64). Additionally, women who conceive successfully after HSG surgery may be at risk of excess iodine, as previous studies showed that women who underwent HSG before pregnancy remained in a prolonged state of high iodine throughout the gestational and postpartum periods (65). Moreover, another study confirmed persistently high levels of iodine after OSCM HSG, and iodine excess did not correlate to improved fertility. However, it was noted that 30% of their population was iodine deficient, and the OSCM HSG corrected that deficiency, which aided in improving fertility for those patients (66). Thus, it is essential to recognize the impact of iodine excess on differing populations, as baseline iodine levels have the potential to influence outcomes of OSCM HSG.

Research on the impact of excess iodine on female reproductive physiology is limited. However, a study by Mahapatra et al. provides some insights. Rats were separated into three groups were given varying levels of iodine in the form of potassium iodide for an extended period of time, and demonstrated that extended exposure to iodine in excess exerted a biphasic mode of action, causing either a hypofunctioning or a hyperfunctioning ovary, with a fertility index of zero at both doses (67). Furthermore, a study evaluating ovarian and uterine histological changes following prolonged iodine excess demonstrated an alteration in the structure and number of ovarian follicles and corpus luteums (68). In addition, uterine changes were also observed which further contributed to the negative alteration of female rat reproductive function (68). Despite current findings, further research is essential to gain a comprehensive understanding of the effects of excess iodine on ovarian structure and function.

### Iodine excess and pregnancy

The physiological demands of pregnancy necessitate an approximately 50% increase in iodine requirements, rendering this population vulnerable to iodine level imbalances (69). A median UIC below 100 μg/L for nonpregnant women and children defines an iodine-deficient population (70). For pregnant women, the WHO defines UIC levels of 150–249 μg/L as adequate iodine status, UIC values below 150 μg/L indicating iodine deficiency, levels between 250 and 500 μg/L being classified as more than adequate, and values exceeding 500 μg/L considered to be iodine excess (70). Iodine deficiency may be exacerbated by avoiding iodine-rich foods, stemming from concerns about heavy metal contamination in these foods, and managing nausea and vomiting (71), so iodine supplementation has become popular. However, pregnant women are at risk of iodine excess due to the intake of water, food, nutritional supplements or additional medications (10). A new study challenges the current WHO recommendation by suggesting a lower limit for iodine intake during pregnancy (72).

**Figure 2:**
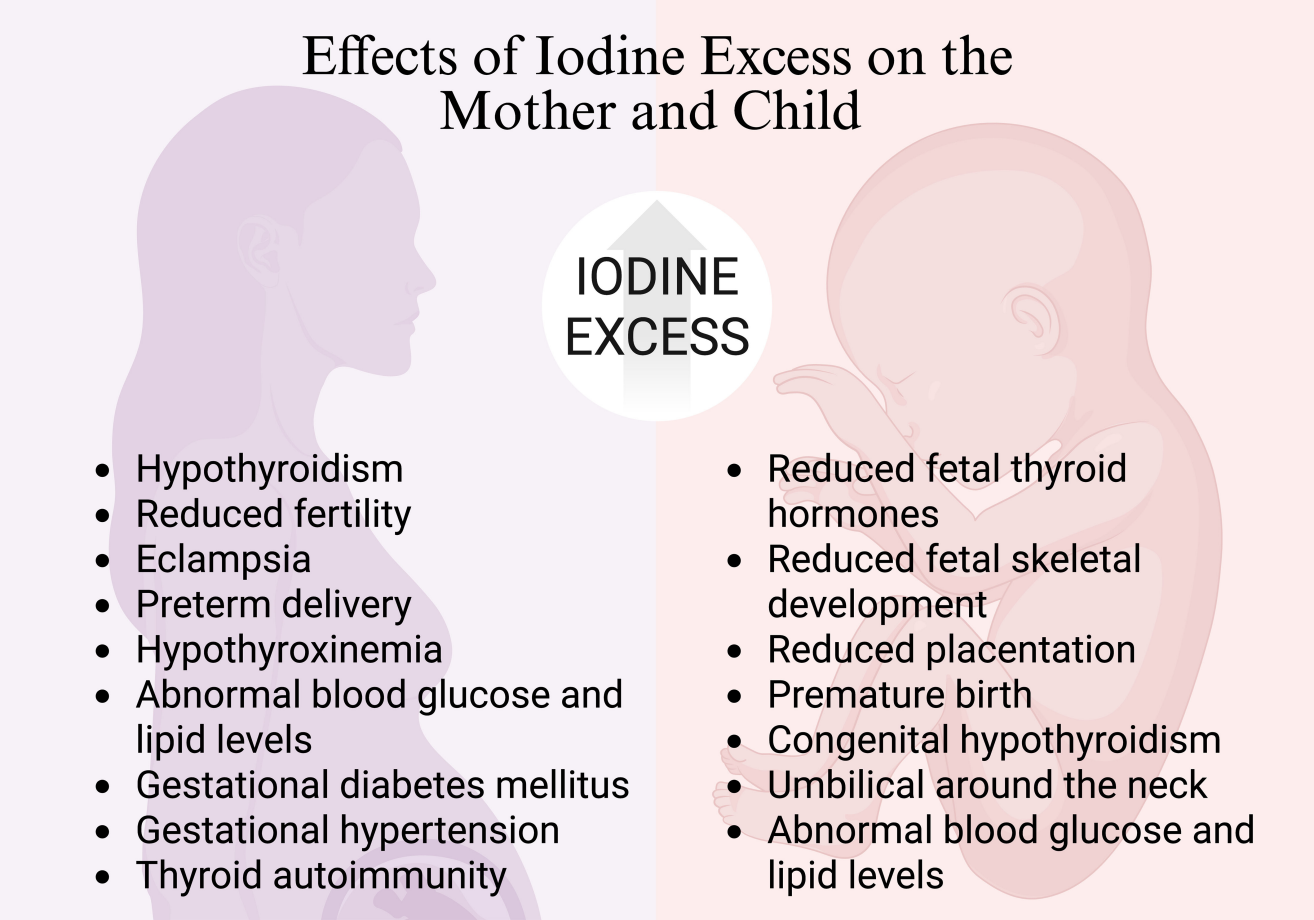
Effects of iodine excess on mother and child. Created with biorender.com.

Excessive iodine during pregnancy can increase serum thyroid-stimulating hormone (TSH) concentrations, negatively impacting maternal thyroid function and potentially leading to further health risks, as seen in **Figure 2**. Findings from an observational study indicate that pregnant women consuming high-dose iodine supplements (>200 μg/day) may have a higher risk of serum TSH elevation compared to with women whose supplemental iodine intake was lower at <100 μg per day (73). Elevated maternal serum iodide can potentially lead to reduced fetal thyroid hormones, fetal skeletal development, placentation and preterm delivery (74). The ingestion of excess maternal iodine tablets during pregnancy has led to several cases of congenital hypothyroidism being reported (75).

Beyond these hormonal effects, excessive iodine intake has also been linked to thyroid autoimmunity, which has been associated with increased infertility rates, polycystic ovary syndrome, premature ovarian insufficiency and endometriosis (76, 77). Excess iodine may trigger immune dysregulation, contributing to autoimmune thyroid diseases such as Hashimoto’s thyroiditis and Graves’ disease, both of which have been associated with excess long-term morbidities in children and adolescence (78). Since immune balance is crucial for implantation, ovarian function and maternal-fetal interactions, these findings suggest that iodine-related immune disturbances could contribute to reproductive challenges (79–81). While direct studies on iodine excess and reproductive immunology remain limited, further research is needed to clarify these potential mechanisms.

In addition to supplements, excess iodine may also be due to diet. For mothers who consumed excessive amounts of seaweed during both pregnancy and lactation, hypothyroidism was diagnosed in their offspring (82, 83). A Finnish study revealed a positive association between elevated serum iodide levels and risk of preterm birth (84). Furthermore, iodine excess during pregnancy was found to be linked to a significant increase in the occurrence of adverse mother and fetus outcomes, including eclampsia, umbilical cord wrapping around the neck, abnormal blood glucose and lipid levels (85).

Moreover, a study involving 214 pregnant women determined that those with UIC between 250-499 µg/L exhibited higher rates of GDM (20.3%) compared to the group with UIC 150-249 µg/L (9.7%) (24). Additionally, HDP, encompassing pre-eclampsia and gestational hypertension, occurred with a prevalence of 33.3% in those with UIC ≥ 250 µg/L compared to 4.3% in those with 150-249 µg/L (24). Neonatal growth parameters, such as head circumference, femur length, and estimated fetal weight were inversely associated with a state of iodine excess (86). Additionally, a cross-sectional study of 7,190 pregnant women in China found that both deficient and excessive iodine intake were associated with an increased risk of maternal hypothyroxinemia, hypothyroidism prevalence and thyroid autoimmunity (87). Despite its clinical significance, there are limited studies concentrating specifically on iodine excess during pregnancy, and maternal and neonatal outcomes. However, with the current research, the potential risks associated with even mild iodine excess during pregnancy warrant careful consideration.

## Endocrine-disrupting chemicals and thyroid function

According to the Endocrine Society, Endocrine-disrupting chemicals (EDCs) are defined as a mixture of chemicals or an exogenous chemical that can interfere with any aspect of hormone action (88). They include a wide range of compounds such as fungicides, plasticizers, pesticides and industrial chemicals, among others (89). Certain EDCs, namely thiocyanate and perchlorate, affect thyroid metabolism by inhibiting the sodium-iodide symporter (90, 91). Additionally, compounds such as flame retardants may disrupt thyroid physiology.

A recent 2024 review examining flame retardants and thyroid function revealed conflicting evidence, with some studies finding a positive association with TSH while others noted a negative one (92). Similar relationships were found between flame retardants and total T3 and total T4 (92). The authors emphasize the need for long-term studies to more conclusively determine the effects flame retardants have on thyroid function (92).

As EDCs affect thyroid physiology, they can potentially impact iodine levels, making them a factor to consider in infertility. As a result, further research into the long-term effects of EDCs on thyroid function, iodine levels and reproductive health is crucial for mitigating possible future fertility challenges worldwide.

## Conclusion

Excess iodine intake is an emerging concern with potential implications for reproductive health. While iodine is essential for thyroid function and overall well-being, an imbalance, whether deficiency or excess, can lead to significant health consequences. This review highlights evidence suggesting that excessive iodine exposure may impair male and female reproductive health by altering semen quality, disrupting hormonal balance, and contributing to conditions such as infertility, GDM, and HDP. Given the rising number of countries experiencing excessive iodine intake due to over-iodization and poor monitoring, it is crucial to implement strategies that ensure a balanced intake. Future research should further investigate the underlying mechanisms and long-term reproductive outcomes associated with iodine excess to refine public health recommendations. By addressing these gaps, we can develop more precise dietary guidelines and policies to safeguard reproductive health while maintaining optimal iodine nutrition.
